# Mesenchymal Stem Cells in Tissue Repair

**DOI:** 10.3389/fimmu.2013.00201

**Published:** 2013-09-04

**Authors:** Amy M. DiMarino, Arnold I. Caplan, Tracey L. Bonfield

**Affiliations:** ^1^Department of Pediatrics, Rainbow Babies and Children’s Hospital, Case Western Reserve University, Cleveland, OH, USA; ^2^Department of Biology, The Skeletal Research Center, Case Western Reserve University, Cleveland, OH, USA; ^3^Department of Pediatrics, Case Western Reserve University, Cleveland, OH, USA

**Keywords:** adult mesenchymal stem cells, anti-inflammatory agents, antimicrobial protein, milieu therapy, regenerative pharmacology

## Abstract

The advent of mesenchymal stem cell (MSC)-based therapies for clinical therapeutics has been an exciting and new innovation for the treatment of a variety of diseases associated with inflammation, tissue damage, and subsequent regeneration and repair. Application-based ability to measure MSC potency and fate of the cells post-MSC therapy are the variables that confound the use of MSCs therapeutics in human diseases. An evaluation of MSC function and applications with attention to detail in the preparation as well as quality control and quality assurance are only as good as the assays that are developed. *In vivo* measures of efficacy and potency require an appreciation of the overall pathophysiology of the model and standardization of outcome measures. The new concepts of how MSC’s participate in the tissue regeneration and wound repair process and further, how this is impacted by estimates of efficacy and potency are important new topics. In this regard, this chapter will review some of the *in vitro* and *in vivo* assays for MSC function and activity and their application to the clinical arena.

## Introduction

Mesenchymal stem cell (MSC) developmental versatility has generated remarkable interest in their potential application in cell-based therapy (1). The primary problematic issue in MSC-based therapy is the variability in outcome (efficacy) and the strength of effectiveness (potency). Once source of variability depends on whether the cells were derived from an allogeneic (different person) or autologous sources (self-derived). Autologous applications are ideal because they eliminate issues of cross-matching, product contamination, or potential for malignancy (2). The down-side of autologous application is availability and the potential for self MSC deficiencies secondary or intrinsic to the disease itself (2). Allogeneic infusions circumvent the issue of possible ineffectiveness but cross-reactivity and longitudinal effectiveness become the concern. Historically, MSCs have been thought of as “immune-privileged” with the lack of MHC II (the classical co-stimulator molecules of the adaptive response) (3). Recently, it has been shown that MSCs can up-regulated MHC II in inflammatory milieus and are, indeed, recognized by the host immune system (4). Whether this is an important component of MSC function *in vivo* or not is a question and may be dependent upon the milieu and anatomical site of action.

The fact that every MSC-donor is different (genetically, physiologically, etc.) clearly becomes the most important of the uncontrolled aspect of both allo- and autologous MSC-based therapies. Published information suggests that the secretion of powerful bioactive molecules by MSCs may differ by a factor of 10 between different donors of matched age and gender (5). Thus, potency in this context might show a high degree of variability. The question remains as to what defines efficacy and how this is related to the potency of the MSC and the overall desired impact clinically of the MSCs *in vivo* in terms of tissue regeneration, wound repair, or immune-modulation. The difficulty arises in the availability of testing MSCs for phenotypic predictors *in vitro*, which would define the *in vivo* ability to function and generate the desired effector responses. This chapter will discuss the current models for the activity of MSCs and how they are correlated with the *in vivo* effectiveness of MSC function and activity.

## Tissue Regeneration and Wound Repair

Mesenchymal stem cells actively respond to stress or injury in a manner that is very similar to how the adaptive and innate immune system cells respond to pathogen exposure or apoptosis. When supplied exogenously, MSCs home to sites of injury primarily inflamed or broken blood vessels. There is an intricate array of soluble mediators generated by MSCs in a milieu specific manner, almost “sensing” the requirement of the environment. These products can promote angiogenesis, regeneration, remodeling, immune cell activation or suppression, and cellular recruitment. At the same time the MSCs can also actively participate in bactericidal activity. MSCs are perivascular cells, pericytes, and as such, they will not respond the same given that the microenvironments of different tissues are not the same, this will also impact efficacy and/or potency. The functionality of the MSC preparation is based upon isolation and expansion procedures and the injury-specific environment that they find themselves in. The ability MSCs to respond to the given environment is attributed to the ability of them to respond to changes or requirements of the milieu through transcriptional regulation and translation of appropriate responding mediators. This responsiveness is due to receptor recognition and signal transduction followed by the release of specific protein mediators that influence the milieu for repair, control of inflammation and infection. The repair process entails regulating extracellular matrix deposition, collagen synthesis, fibroblast proliferation, platelet activation, fibrinolysis, and angiogenesis. The immune process often involves suppressing T-cells, activating macrophages, and potentially recruiting neutrophils. Studies have also suggested that immune regulation by the MSCs is defined by the soluble products generated by the MSCs facilitating changes in immune cell activation status. The products produced by the MSCs are not different from those of the immune system and can include cytokines, inflammatory mediators, and antimicrobial proteins. Table 1 lists some of these products and references of production and action.

**Table 1:** **Mesenchymal stromal cells and paracrine factors of inflammation**.

Mediator	Function	Reference
TNFα stimulated gene 6 (TSG-6)	Binds hyaluronan, inhibits inflammatory functions	(58, 59)
IL-1RA	Blocks IL-1α and IL-1β function in macrophage activation, T-cell recruitment	(60–62)
PGE_2_	T-cell suppression, decrease IL-10 production. Suppresses IL-17 production	(63–65)
Indoleamine 2,3-dioxygenase	T-cell suppressor	(66–68)
Nitric oxide	Pro-versus, anti-inflammatory, macrophage regulator. T-cell suppressor	(69–71)
TGFβ1	Suppress T-cell activation, macrophage activation. Inhibits dendritic cell migration	(72 –74)
SDF-1 (stromal cell derived factor)	Stem cell progenitor cell recruitment, tissue regeneration	(75–78)
VEGF, EGF, IGF-1	Anti-apoptosis, differentiation, and angiogenesis	(79–81)
LL-37	Antimicrobial and anti-inflammatory functions	(38, 82)

## Efficacy

**Figure 1:**
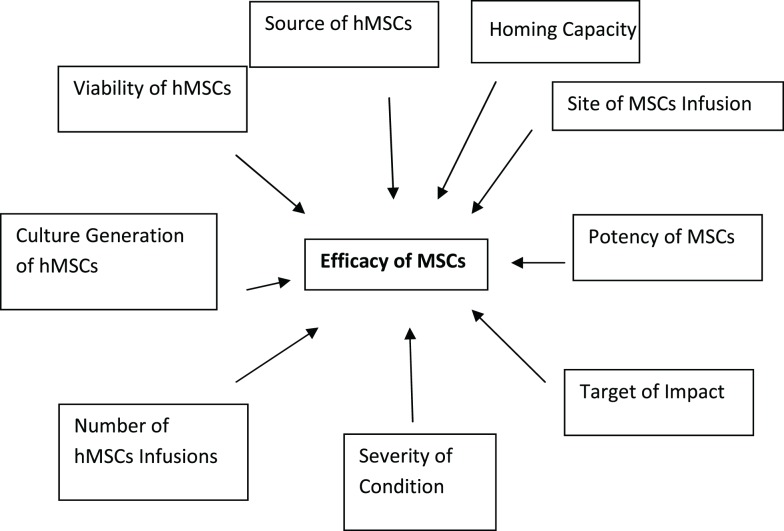
**The dynamic definition of MSC efficacy**. Efficacy or the biological impact of MSC on biological processes is dependent on a variety of parameter. As outlined in the schematic below the variability in efficacy is dependent not only on the source and quality of the MSCs themselves but also on the response of the recipient to the administration of MSCs. This is a dynamic and continuous process that can ultimately be defined by the potency of the MSCs in the context of disease, model, and outcome measure.

What is efficacy? Efficacy as defined as “the power to produce an effect” (6). The “effect” in the context of MSC biology is whether the end-point is reconstruction, regeneration, repair, anti-inflammatory, anti-autoimmunity, or any other hypothesized clinical applications for the use of MSCs clinically. To further complicate the notion of MSC efficacy is the focal point of differentiation and desired function of the MSCs. There is also a distinction between the efficacy of cell–cell impact and the efficacy of soluble products generated by MSCs. The required numbers of infusions of MSCs may also confound efficacy. In a study evaluating graft versus host disease (GvHD), it was found that in some of the patients, several infusions were required to sustain the positive outcome (7, 8). This does not imply that all of the GvHD subjects responded in a positive manner to the MSC load, some did not which begs the question of potency. Is the fact that some patients require more dosages than others due to the potency or “strength of the MSC preparation” and is the “strength of the MSC preparation” also defined by the degree of injury or illness and the general health and age of the recipient? These are all very difficult matters to control in the context of clinical trials and may have a major role in some of the complexity of the outcomes in MSC-based clinical trial applications. Figure 1 show efficacy being defined by a variety of factors which are not only associated with the MSCs themselves but also the host involvement in defining the ultimate residence and milieu for MSC effectiveness (9, 10).

## Potency

Potency, or the ability or capacity to achieve or bring about a particular result, is a major component which defines the ultimate efficacy or the power to achieve a particular event (11). The difference between these two terms, potency and efficacy, lies in the generalized capacity to be impactful (potency) and with what intensity is this impact instituted (efficacy). MSCs must first have the capacity to achieve the desired effect, which then is followed by how much and how long MSCs need to be in the desired context to institute the response. The question also begs the issue of what is the desired response? In the asthma studies, when MSCs are infused in the absence of inflammation, the tendency is to have a greater cellular recruitment into the lung (suggesting a xenographic response) (6, 12). However, when the hMSCs are given in the context of airway disease (both allergen challenge or infection), they then become immunosuppressive reversing the cellular recruitment, which is generally seen in response to the pulmonary insult. In these studies, the hMSCs were given intravenously at 10^6^ cells/mouse post-challenge. The difference between these observations has to do with the localization of inflammation and injury in the context of allergen challenge. These studies further showed that the MSCs are potent in the context of pre-existing airway disease. Similarly, the intensity by which the MSCs induced the response is shown by the variability of the change in lung differential between study sets. The investigators in these studies further define this variability based upon the separately assayed ability of the MSCs to induce bone formation in an *in vivo* implantation efficacy assay. These studies showed that the assay for bone formation mirrored the *in vivo* decrease in cellular recruitment even in the context of allergen challenge. The connection between bone formation and lung efficacy is not obvious to us and likely somehow reflects the functionality of MSCs in two different microenvironments.

## Measures of Efficacy

Traditional measures of efficacy have been done *in vitro* using very tightly controlled environments in terms of medium, fetal calf serum (FCS), supplements, and buffering systems (12, 13). In some cases, the specific serum lots are essential for the optimal biologic response of the model system. The idea behind the *in vitro* modeling is the ability to take the multipotent MSCs and treat them with the conditions for effective differentiation. The end-point activation or differentiation is then tightly controlled for validation of the multipotent differentiation process. The issues of pre-phenotype before differentiation and post-phenotype after differentiation can be different from one laboratory to another, which confounds the terms of “efficacy” when referring to MSCs. Keep in mind that as pericytes *in vivo*, MSCs function in response to injury. This is quite different and separate from their capacity to differentiate into bone, cartilage or other surrounding tissues in an *in vitro* culture environment. Commercialization would require these processes of activation or differentiation to be standardized so that preparations could be compared across institutions and continents. Further, the *in vitro* scenario which is chosen is often the one used in the context of the application being studied. It would be useful to probe the question as to the differences in the differentiation potential of hMSCs in the context of a specific application of choice. For example, if the application is for asthma, would it be appropriate to use the *in vitro* chondrocyte differentiation process as well as adipocyte differentiation to define *in vitro* efficacy and how this correlates to the observations *in vivo*? These *in vitro* models for identifying the capacity for MSCs multi-lineage differentiation use different differentiation conditions to achieve chondrocytes, adipocytes, and osteoblasts (2, 13, 14). Alternatively, studies have also focused on the functional ability of the MSCs to induce an effect such as suppression of T-cell activity and cytokine production (15, 16).

### Differentiation efficacy

**Figure 2:**
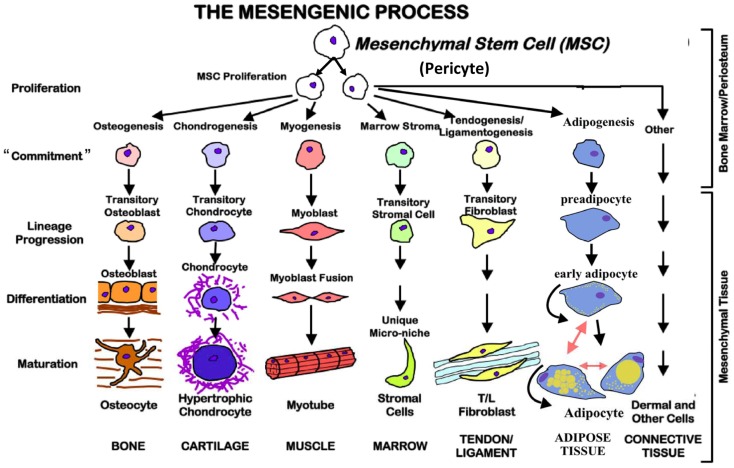
**The mesengenic process**. Mesenchymal stem cells are multipotent and possess the ability to proliferate and commit to different cell types based on the environmental conditions. They also may be redirected from one lineage to another.

Mesenchymal stem cells, regardless of where they are isolated from, can be induced to differentiate *in vitro* in an effort to define efficacy. As stated, efficacy is defined by the end-point differentiated product, which by its nature is defined by the conditions of differentiation (17). There is no quantitative way in which MSCs can be defined from the beginning as to their ability to differentiate into either one of the following types of end-point phenotypes. To complicate things further, just because a cell is pushed into a specific direction of differentiation, it appears that they have the capacity to be redirected. For example, an epidermal skin cell can be pushed into another type of cell completely depending upon the conditions of culture. The basis for the ability to differentiate and to re-direct to accommodate the milieu or teleological “sensing” of required response is based upon the identification or the originating cellular source of the MSC. MSCs are thought to be derived from pericytes (18). Pericytes are perivascular cells with multifunctional activities which are just now being elucidated. The functional interaction of pericytes with the endothelial cells is thought to be the source for differentiation and definition of MSC differentiation (19). Injury results in vascular changes even at the micro-vascular level. These changes stimulate pericyte differentiation into targeted MSCs that hone to the injury and repair perturbed tissue as well as modulate the surrounding *in vivo* environment. As pericytes are multipotent their ability to define MSCs targeted function is outlined in Figure 2. The environment of culture *in vivo* ultimately defines the process of differentiation and end-point tissue. Variations of the tissue differentiation will depend on the quality and the quantity of specific inducers of the differentiation process. The traditional measures of MSC differentiation post-transition from the pericyte include the following.

#### Chondrocytes

For chondrocyte differentiation, MSCs are put into spheroids by centrifugation and incubation in chondrogenic-specific induction medium using a chemically defined medium of DMEM containing specific lots of FCS and transforming growth factor beta (TGF-β) for 25 days (20–22). To define the success of chondrocyte development, the spheroids are fixed in 10% buffered formalin for 2 h at room temperature followed by dehydration with xylene and paraffin. Paraffin sections then are stained with 0.1% Safranin O to detect mucopolysaccharide synthesis indicative of chondrocyte production. An alternative to the histology, some investigators use quantification of glycosaminoglycans (GAG) to determine differentiation potential.

#### Adipocytes

For adipocyte differentiation, MSCs are cultured in a defined medium including FCS, dexamethasone, ascorbic acid, and 1-methl-3-isobutyl-xanthine. Growth occurs for at least 14 days followed by Oil-Red O staining to show adipocyte differentiation (23, 24).

#### Bone

For osteoblastic differentiation, MSCs are cultured in DMEM supplemented with 10% FBS, β-glycerophosphate, dexamethasone, and ascorbic acid. After 21 days, the cells can be stained with van Kossa’s stain to reveal osteogenic differentiation or chemically analyzed for calcium accumulation (25–27).

### Functional efficacy

A variety of functional assays can also be used to measure MSC efficacy. The point of these assays is to measure the ability of the MSCs to induce a specific effect. MSCs have been shown to have an immunosuppressive effect on T-cells (15) both as an activity and also in their ability to produce lymphokines (28). Traditional T-cell proliferation assays using the incorporation of tritiated thymidine 3 days post stimulation in the presence of antigen presenting cells with and without MSCs can be an easy 3-day assay for MSC efficacy. These studies can be further simplified by using one of the newer assays that involve using T-cell ATP levels as an indicator of activity (29). In these studies, no radiolabel is required and the assay is a direct 18-h output. T-cell cytotoxic cells can also be used as indicators of MSC efficacy for the same purpose as the T-cell activity assay (30, 31). The interesting issue is the differences between the assays in terms of MSCs effector function: the MSC preparation which suppresses the T-cell proliferative responses may not be as effective at attenuating the ability of T-cells to mediate a cytotoxic response.

## *In vivo* Versus *In vitro* Models

Several *in vivo* experimental studies have shown the value of allogeneic or xenogenic MSCs for a variety of disorders including: sepsis, hepatic failure, acute renal failure, and myocardial infarction (32). The issue with *in vivo* models of efficacy has to do with the validity of the model itself and the inherent variability that exists with *in vivo* experimentation. The other issue that needs to be addressed is the assessment of efficacy and the duration required to obtain that outcomes. *In vivo* models are more expensive and are inherently more time consuming with the issue of translation to human applications a major focus. The choice of model, duration of MSC exposure, dosing required to induce the effect, and final outcome can be interpreted as strong indicators of the ability to use MSCs *in vivo*. Interestingly there have been a variety of studies in which xenographic hMSCs have been utilized in animal models that show similar effectiveness to allogenic and autologous MSCs (6, 12). In acute renal failure in mice, intravenous MSC therapy hastened renal tubular epithelial cell recovery (4), and markedly improved mortality from peritoneal sepsis in mice regardless of whether the MSCs were allogeneic or autologous (33). For example, the documented studies involving lung inflammation, administration of MSCs was post-injury suggesting the injury requirement in the function (recruitment or activation) of the MSCs to the interface of lung injury. These studies would provide *in vivo* models of efficacy and would define the potential for different “efficacy” and “potency” directives depending on the injury itself. A few examples of specific models will be discussed.

### Lupus

Mesenchymal stem cells have been shown to have a profound impact on the impact of disease progression in the murine model of lupus (34). Murine lupus can be induced in 6- to 7-week-old female C57BL6 mice by immunization with an emulsion containing 300 μg of purified myelin oligodendrocyte glycoprotein (MOG) and an equal volume of complete Freund adjuvant followed by intra-peritoneal injections with *Bordetella pertussis*. Animals are scored daily for neurological symptoms according to the murine lupus clinical severity scale: 0 = asymptomatic; 1 = partial loss of tail tonicity; 2 = tail paralysis; 3 = hind limb weakness; 4 = hind limb paralysis; 5 = limb paralysis; 6 = death. MSCs have been given intravenously at 10^6^ MSCs/100 μL (using saline as the vehicle control). Changes in the scoring of the MSC-treated mice can then be used as a measure of efficacy. For potency, the same model can be used comparing single dosing versus multiple dosing for selected time points (35, 36).

### Sepsis

Mesenchymal stem cells have been shown to be effective at improving survival of mice in the sepsis model (37). Sepsis can be induced in C57Bl/6J mice by cecal ligation and puncture, followed 6 h later by an intravenous injection of MSCs or saline. Bronchoalveolar lavage (BAL) fluid and tissues can be collected for analyses at 24 h post infection. MSC treatment has been shown to significantly reduced mortality in septic mice receiving appropriate antimicrobial therapy which included reduced systemic and pulmonary cytokine levels in mice with induced sepsis, preventing acute lung injury and organ dysfunction (38).

### Acute lung injury

In this model, mice are given and intratracheal instillation of lipopolysaccharide with and without MSC (39). Total cell count, cytokines associated with the lung injury, and other pathophysiological data can be assessed and correlated with MSC impact on LPS-induced lung injury. This model would be relative to the MSCs ability to react versus host response and bacterial burden. The advantage of this model is that it is fast, and reproducible. The disadvantage is that it focuses on only the trophic components of MSCs efficacy (37).

### Acute asthma

This is an immunological model and would be used to define the suppressive effects of the hMSCs (40). In these studies, Balb/c mice can be sensitized by intra-peritoneal injections of ovalbumin (OVA) emulsified in Al(OH)_3_. After 14 days are then exposed to 1% weight/volume OVA in sterile saline by aerosolization every day for 5 days (12). hMSCs are given on either day 14 or day 16 by tail vein injection with 1 × 10^6^ hMSC/mouse in 100 μL of PBS. As a positive control for immunosuppression, a subset of the OVA sensitized, OVA challenged mice received dexamethasone at 10 mg/kg. The dexamethasone can significantly decrease total cell recruitment and eosinophil counts and can be used for a control of immunosuppression.

### Multiple sclerosis

In the autoimmune model of multiple sclerosis (MS), mice are injected with myelin protein to induce experimental autoimmune encephalitis EAE (34). The animals mimic many of the pathophysiological processes associated with MS development and progression. Infusion of MSCs in the EAE model of MS significantly improved survival and decreased markers of inflammation associated with specific outcome of disease (41). The resolution of EAE definable markers of pathology can be a powerful tool for measuring *in vivo* efficacy and potency of MSCs.

There are other suggested *in vivo* models such as hepatic failure, kidney failure, and myocardial infarction but these require more time, are even more expensive, and at the end, probably do not provide any more detail than the short term *in vivo* efficacy assay described above accept for the end-point evaluation of investigation (42, 43). Depending on the perspective, a focused approach to the type of outcome desired and the criteria of that selection may be important in choosing any of these models whether they are simple or complicated.

### Ex vivo

With the significance in translating between the observations of *in vivo* modeling and *in vitro* modeling in hMSC therapeutics, some groups have been able to develop *ex vivo* models using perfused tissue from humans post-surgery. Matthay and co-workers adapted an *ex vivo* perfused human lung preparation for studies of acute lung injury to test the effects of MSC therapy (39). In this study, the right middle lobe was injured with intra-alveolar endotoxin, resulting in a sharp increase in lung endothelial and epithelial permeability to protein leading to pulmonary edema and loss of the normal capacity of the alveolar epithelium to remove alveolar fluid. When the lung was treated 1 h after instillation of the endotoxin with intra-bronchial allogeneic MSC, the lung vascular permeability and extra-vascular lung water returned to normal levels. These studies also used culture medium generated from MSCs induced the same effect suggesting the impact was based upon soluble response of the MSC to the lung milieu. Interestingly, investigators using the EAE model also showed that the impact of MSC on EAE pathophysiology was mediated through products generated by the MSCs themselves in response to the inflammation (44).

### Conundrum of controls *in vivo* MSC response

One issue that is often brought up is the issue of cellular specificity in terms of response to MSCs. What is the appropriate control of MSCs administration *in vivo*? The control would need to represent the species of origin and phenotypic presentation. This is a major issue since in many studies the MSCs are of human origin and are not autologous. These are xenographic and cross-species studies. Although, the MSCs are thought to be immune-privileged, the cross-species is a good control for the species effect. In other situations, non-MSC-based cells are used as controls such as fetal fibroblasts or bone marrow-derived differentiated cells or fibroblasts. These might be derived from the same species, but they will not have the same phenotypic presentation since it is likely that they will have MHC class II and maybe even MHC class I expression.

## Delivery, Timing, and Sources of MSC Therapy

### Route of delivery

The optimal route of delivery for the MSC is unknown (44, 45). Would intravenous delivery of MSCs result in the same benefit as direct tissue delivery? The main point is whether the MSCs have the capacity to home or to direct “themselves” to injured organs and whether the injury occurs in the liver, kidney, or the lung. The other possible context is that it is the homing issue as it is in the accessibility of the injury sites to the vasculature and the “pericyte” nature of the MSCs themselves. Systemic administration allows for the process of journey and differentiation, but may impact the ultimate efficacy and potency of the MSC preparation. The benefit of direct tissue administration is the milieu effect and optimization of the MSC preparation.

### Dosing and timing of treatment

The dosing and timing of MSC treatment is also an issue that still needs to be addressed and confirmed for translational applications. In the context of a specific disease entity, dosing may be defined by the potency, efficacy of the MSC preparation, and the status and phenotype of the disease. In the *in vivo* asthma studies, a single dosing of MSC was used and showed significant impact on the murine model of both acute and chronic disease (6, 12). This is the same for the other *in vivo* models (34, 46, 47). Interestingly in clinical trials of MSCs in Crohns disease, osteogenesis imperfecta, MS, and acute steroid-refractory GvHD, multiple infusions were required to sustain or induce the clinical improvement (17, 48). In these clinical studies, the definition of who received multiple dosages, why they received multiple dosages, and were these from the same batch of MSCs of different donors is an issue. This makes understanding the biological components and success of the studies difficult. Since some patients required more infusions than others, suggests and efficacy and potency effect. Future studies need to better control for the efficacy and potency within the clinical trial using both *in vivo* and *in vitro* assessments of activity. This would be a costly endeavor but essential as studies translate into the application to human disease and the cost of each MSC preparation.

### Targeting blood bore MSCs

With the notion that pericytes are stimulated to define MSC function and homing properties, the obvious application is providing targeting for directed MSC function *in vivo* (49, 50). For example, studies have used genetically engineered MSCs using *ex vivo* retroviral transduction to overexpress the pro-survival gene *Akt1* (encoding the Akt protein which is important for survival) to improve overall survival of MSCs *in vivo* (51). Transplantation of MSCs overexpressing Akt into ischemic rat myocardium inhibited the process of cardiac remodeling by reducing intramyocardial inflammation, collagen deposition, and cardiac myocyte hypertrophy. MSCs transduced with *Akt1* restored fourfold greater myocardial volume than equal numbers of cells transduced with the control reporter gene. These observed effects were dose (cell number) dependent. Thus, MSCs genetically enhanced with *Akt1* can repair infarcted myocardium, prevent remodeling, and nearly normalize cardiac performance. Other studies have started to focus on the use of targeted MSC in other diseases. In one set of studies, an adeno-associated virus vector was used to disrupt dominant-negative mutant *COL1A1* collagen genes in MSCs from individuals with the brittle bone disorder osteogenesis imperfecta (52). These studies demonstrated that the concept of MSC correction (53) and potential for directed gene therapy can be successful in human stem cells providing exciting potential for therapeutic applications. Further, in recent studies, glyco-engineering of hematopoietic cell E-/L-selectin ligand (HCELL) on MSCs can target hMSCs to marrow, licensing transendothelial migration without chemokine signaling via a VLA-4/VCAM-dependent pathway (54, 55). This homing receptor concept also has the potential of introducing new ways of specifically targeting cell-based therapy to disease sites.

### Sources of MSCs

Mesenchymal stem cells can be extracted from several sources within the body and their usefulness in different disease processes may depend on their source. Examples of sources include adipose and synovial tissue, peripheral blood, skeletal muscle, umbilical cord blood, placenta, and bone marrow. The procedure for obtaining MSCs from donors can be relatively invasive (i.e., bone marrow aspiration) or minimally invasive (i.e., liposuction). Although MSCs obtained from different regions of the body show similar potential for differentiation and therapeutic effects, they each express different cellular markers. For example, adipose-derived MSCs express higher levels of CD49d, CD34, and CD54 whereas bone marrow-derived MSCs have higher levels of CD106 (56, 57). The clinical and therapeutic significance of these markers remains to be elucidated, however. Also, the differentiation potential of MSCs may vary from source to source, and number of actual cells obtained from each source may vary based on their source, age of the donor, and co-morbid conditions. More in depth studies are needed to determine if certain MSC sources are more beneficial in certain diseases and if their therapeutic effectiveness and safety profiles are similar.

## Conclusion

Mesenchymal stem cells have become a major focus for a potential resource in therapeutic cell-based therapies. MSCs are multipotent cells derived from stromal tissue, which have the capacity to differentiate into mesodermal and endodermal types of cells. Not only do MSCs have the capacity to differentiate into different types of cells depending on the tissue matrix, they also actively contribute to their milieu by secreting soluble products that actively participate in MSC and surrounding cell phenotype. The issues related to therapeutically translating MSCs into the clinical arena are based upon the ability to predict the host response, the intensity of the response, and the duration of the response. These parameters are associated with the potency and efficacy of the stem cell preparation. *In vitro* and *ex vivo* assays are available to define the relative efficacy of several *in vitro* and *in vivo* assays which can ultimately be used to define MSC efficacy and potency, but these are going to be tissue-specific and disease-specific. In the end, correlating a specific type of disease process (inflammation versus remodeling) must be used to define the activity of the MSCs. This will become especially true as new sources besides bone marrow-derived MCSs become clinically available and are approved for clinical applications.

## Conflict of Interest Statement

The authors declare that the research was conducted in the absence of any commercial or financial relationships that could be construed as a potential conflict of interest.
